# Vapochromic Behaviour of M[Au(CN)_2_]_2_-Based Coordination Polymers (M = Co, Ni)

**DOI:** 10.3390/s120303669

**Published:** 2012-03-16

**Authors:** Julie Lefebvre, Jasmine L. Korčok, Michael J. Katz, Daniel B. Leznoff

**Affiliations:** Department of Chemistry, Simon Fraser University, 8888 University Drive, Burnaby, BC V5A 1S6, Canada; E-Mails: jlefebvr@ucalgary.ca (J.L.); jkorcok@tru.ca (J.L.K.); mjkatz@sfu.ca (M.J.K.)

**Keywords:** vapochromism, cyanide, X-ray structures, cobalt(II), nickel(II)

## Abstract

A series of M[Au(CN)_2_]_2_(analyte)_x_ coordination polymers (M = Co, Ni; analyte = dimethylsulfoxide (DMSO), N,N-dimethylformamide (DMF), pyridine; x = 2 or 4) was prepared and characterized. Addition of analyte vapours to solid M(μ-OH_2_)[Au(CN)_2_]_2_ yielded visible vapochromic responses for M = Co but not M = Ni; the IR ν_CN_ spectral region changed in every case. A single crystal structure of Zn[Au(CN)_2_]_2_(DMSO)_2_ revealed a corrugated 2-D layer structure with *cis*-DMSO units. Reacting a Ni(II) salt and K[Au(CN)_2_] in DMSO yielded the isostructural Ni[Au(CN)_2_]_2_(DMSO)_2_ product. Co[Au(CN)_2_]_2_(DMSO)_2_ and M[Au(CN)_2_]_2_(DMF)_2_ (M = Co, Ni) complexes have flat 2-D square-grid layer structures with *trans*-bound DMSO or DMF units; they are formed via vapour absorption by solid M(μ-OH_2_)[Au(CN)_2_]_2_ and from DMSO or DMF solution synthesis. Co[Au(CN)_2_]_2_(pyridine)_4_ is generated via vapour absorption by Co(μ-OH_2_)[Au(CN)_2_]_2_; the analogous Ni complex is synthesized by immersion of Ni(μ-OH_2_)[Au(CN)_2_]_2_ in 4% aqueous pyridine. Similar immersion of Co(μ-OH_2_)[Au(CN)_2_]_2_ yielded Co[Au(CN)_2_]_2_(pyridine)_2_, which has a flat 2-D square-grid structure with *trans*-pyridine units. Absorption of pyridine vapour by solid Ni(μ-OH_2_)[Au(CN)_2_]_2_ was incomplete, generating a mixture of pyridine-bound complexes. Analyte-free Co[Au(CN)_2_]_2_ was prepared by dehydration of Co(μ-OH_2_)[Au(CN)_2_]_2_ at 145 °C; it has a 3-D diamondoid-type structure and absorbs DMSO, DMF and pyridine to give the same materials as by vapour absorption from the hydrate.

## 1. Introduction

Vapochromic materials, which display colour (optical absorption) or luminescence changes upon exposure to vapours of volatile organic compounds (VOCs), are of great interest due to their potential applications for chemical vapour detection [1–16]. Such sensors are commercially important as environmental monitoring devices, as safety systems in industrial settings and for defence and security applications [17]. As an example of a vapochromic material, the polymeric Prussian Blue-type Co^2+^/[Re_6_Q_8_(CN)_6_]^4−^ (Q = S, Se) material yields dramatic changes in the visible spectrum when exposed to specific VOCs; the colour changes are linked to the sensed solvent impacting the geometry and hydration around the Co(II) centres [12]. A range of compounds based on Au(I), Pd(II) and Pt(II) coordination polymers have also been extensively investigated for their vapochromic responses [1–12,14]. The vapochromism in these systems is based on changes in both the visible absorption and emission spectra. In the linear {Tl[Au(C_6_Cl_5_)_2_]}_n_ polymer, weak interactions between the Tl atoms and the absorbed VOC molecules modify the emission spectra [8]. On the other hand, emission changes in [Pt(CN-R)_4_][M(CN)_4_] (R= *iso*-C_3_H_7_ or C_6_H_4_-C_n_H_2n+1_; n=6, 10, 12, 14 and M = Pt, Pd) occur when metal-metal distances are modified due to the presence of VOC molecules in lattice voids [9,10,18]. A related mode of action is operative for supramolecular columns of Au_3_(MeN=COMe)_3_ molecules, in which VOCs intercalate or otherwise impact the stacking of one polymorph, thereby altering the emission properties [1–3,19,20]; similarly, crystalline, luminescent {Au_3_(*p*-MeC_6_H_4_N=COEt)_3_}_2_ becomes non-emissive upon exposure to hexafluorobenzene [7].

Among coordination polymers, cyanometallate building blocks have been particularly heavily utilized to form extended networks [21–25]. Despite this, [Au(CN)_2_]^−^ has been relatively neglected as a building block in coordination polymers despite its unusual features: it is linear, forms gold-gold intermolecular interactions, and is luminescent [26–34]. We have reported the new vapochromic Cu(μ-OH_2_)_2_[Au(CN)_2_]_2_ and Zn[Au(CN)_2_]_2_ coordination polymer materials [35–38]. The Cu(μ-OH_2_)_2_ [Au(CN)_2_]_2_ system reacts reversibly with certain VOCs containing nitrogen- or oxygen-donors (or irreversibly with NH_3_) in the solid-state, generating significant analyte-specific colour changes, as well as distinct changes to the number and position of the ν_CN_ IR bands. An examination of the crystal structures of the dimethylsulfoxide (DMSO), N,N-dimethylformamide (DMF) and pyridine adducts allowed a postulation of the mechanism of reversible vapochromism: the Cu[Au(CN)_2_]_2_ superstructure flexibly distorts to accommodate analyte binding to Cu(II); this binding changes the ligand field, thereby impacting the colour. Changes in the cyanometallate superstructure can also be transmitted via the ν_CN_ pattern [35,36].

Substitution of copper(II) for zinc(II) yields four polymorphs of Zn[Au(CN)_2_]_2_ [37,38], which are highly emissive upon UV-excitation and, more importantly, are extremely sensitive to ammonia vapour; exposure causes a large emission shift [37]. As in the Cu(II) system, ammonia binds to the Zn(II) centre, which triggers the flexible Zn[Au(CN)_2_]_2_ superstructure framework to dynamically adapt to accommodate the analyte. This changes the gold-gold distances in the structure and, since these aurophilic interactions are the source of the emission [30–32], any change in their geometry or topology impacts the emission energy. Both the Cu(II) and Zn(II) coordination polymers are oxygen-stable and thermally robust.

It is clear from the mode of action that the ligand coordination preferences of the metal cation (Cu or Zn) are a key feature influencing which analytes are ultimately sensed, and which are more favoured from a sensitivity perspective [39]. Thus, examining other metal cations as the binding site is worthwhile both as a comparison to the copper(II) and zinc(II) systems and in the possibility of preparing improved sensor materials or a sensor for new analytes that would not be significantly detected by the vapochromic Cu(II) or luminescent Zn(II) cyanoaurate systems.

Towards this end, we recently reported the isostructural M(μ-OH_2_)_2_[Au(CN)_2_]_2_ series, where M = Mn, Fe, Co, Ni and Cu [40,41]. The Cu(II) analogue shows strong, sensitive vapochromic responses to a series of analytes, as described above [35,36]. This manuscript examines the vapochromic response of the isomorphous Co(II) and Ni(II) analogues to a series of analytes—DMF, DMSO and pyridine—which were studied in detail for the Cu(II) system and which showed moderate sensitivity, thereby allowing for a direct comparison of vapochromic efficacy and superstructural flexibility as a function of metal cation. A qualitative report on the vapochromic response of Co[Au(CN)_2_]_2_ with a series of analytes and their IR shifts was very recently reported, but was lacking in any structural or other characterization data on the Co-analyte system or the parent compound [42,43].

## 2. Experimental Section

### 2.1. General Procedures

All manipulations were performed in air unless otherwise noted. All reagents were purchased from commercial sources and used as received, except for M(μ-OH_2_)[Au(CN)_2_]_2_ (M = Co, Ni), which were prepared as previously described [40,41].

CAUTION: Although we have experienced no difficulties, perchlorate salts are potentially explosive and should be used in small quantities and handled with care.

Infrared spectra were recorded on a Thermo Nicolet Nexus 670 FT-IR spectrometer with samples prepared as KBr pressed pellets or analyzed with a Pike Miracol ATR attachment (Table 1). The resolution of the instrument was set at 1 cm^−1^ and the spectra were collected between 400 (KBr) or 700 (ATR) and 4,000 cm^−1^. Microanalyses (carbon, hydrogen and nitrogen) were performed at Simon Fraser University using a computer-controlled Carlo Erba (Model 1110) CHN analyzer. Thermogravimetric analyses (TGA) of all complexes were performed in an air atmosphere on a Shimadzu TGA-50 instrument at a rate of 5 °C per minute. Solid-state UV-Vis-NIR absorption spectra were measured by reflectance using an Ocean Optics SD2000 spectrophotometer equipped with tungsten halogen and deuterium lamps. Prior to analysis, the samples were ground into fine powders and placed on a glass plate. Magnesium oxide powder, prepared in the same fashion, was used as a reference. Powder X-ray diffractograms were collected on a Rigaku RAXIS-Rapid Auto diffractometer equipped with a Cu K_α_ source (λ = 1.54056 Å). Samples were mounted on a glass fiber using grease and were exposed, as the phi axis was spinning (10° s^−1^), for a period of 20 to 60 minutes (with the chi axis fixed at 0 and omega fixed at 90°).

### 2.2. Synthesis of Zn[Au(CN)_2_]_2_(DMSO)_2_

To a DMSO solution (0.5 mL) of Zn(ClO_4_)_2_ (32 mg, 0.086 mmol), a DMSO solution (0.5 mL) of K[Au(CN)_2_] (50 mg, 0.17 mmol) was added dropwise. The solution was allowed to evaporate slowly. Crystals of Zn[Au(CN)_2_]_2_(DMSO)_2_ were formed over several days of slow evaporation. Yield: 0.030 g, 42%. Anal. Calcd. for C_8_H_12_N_4_Au_2_O_2_Zn: C, 13.35%; H, 7.79%; N, 1.68%. Found: C, 13.50%; H, 8.04%; N, 1.72%. IR (KBr): 3,009 (w), 2,919 (w), 2,186 (m, ν_CN_), 2,175 (m, ν_CN_), 1,314 (w), 1,299(w), 1,030 (m), 1,013 (s, ν_S=O_), 1,005 (s, ν_S=O_), 957 (m) cm^−1^.

### 2.3. Synthesis of Co[Au(CN)_2_]_2_(DMSO)_2_

Co(*μ*-OH_2_)_2_[Au(CN)_2_]_2_ (0.125 g, 0.211 mmol) was mixed with DMSO (1 mL) and left to sit for 72 hours, covered. The resulting pink fine powder was then isolated by decantation and left to dry in ambient air. The composition was found to be Co[Au(CN)_2_]_2_(DMSO)_2_. Yield: 0.121 g, 80.4%. Anal. Calcd for C_8_H_12_N_4_Au_2_CoO_2_S_2_: C, 13.47%; H, 1.70%; N, 7.86%. Found: C, 13.51%; H, 1.73%; N, 7.69%. IR (KBr): 3,021(w), 3,009(w), 2,920(w), 2,177(s, ν_CN_), 1,421(w), 1,408(w), 1,316(w), 1,298(w), 1,031(m), 997(s), 957(m), 938(m), 905(w), 717(w), 467(w), 441(w) cm^−1^. The same product can also be obtained by vapour absorption of DMSO by Co(μ-OH_2_)_2_[Au(CN)_2_]_2_ or Co[Au(CN)_2_]_2_.

### 2.4. Synthesis of Ni[Au(CN)_2_]_2_(DMSO)_2_

A DMSO solution (0.75 mL) of Ni(NO_3_)_2_·6H_2_O (0.057 g, 0.20 mmol) was added to a DMSO solution (1.75 mL) of K[Au(CN)_2_] (0.123 g, 0.427 mmol). A turquoise blue powder started to precipitate after approximately one hour. It was collected by filtration 24 hours later and air-dried. The composition of the final product was determined to be Ni[Au(CN)_2_]_2_(DMSO)_2_. Yield: 0.101 g, 70.8%. Anal. Calcd. For C_8_H_12_N_4_Au_2_NiO_2_S_2_: C, 13.48%; H, 1.70%; N, 7.86%. Found: C, 13.70%; H, 1.81%; N, 8.10%. IR (KBr): 3,434(m), 3,010(w), 2,919(w), 2,189(s, ν_CN_), 2,180(s, ν_CN_), 1,629(w), 1,409(w), 1,314(w), 1,299(w), 1,032(m), 1,008(s), 1,001(s), 956(m), 713(w), 480(w), 430(w) cm^−1^.

### 2.5. Synthesis of Ni[Au(CN)_2_]_2_(DMF)_2_

Ni(*μ*-OH_2_)_2_[Au(CN)_2_]_2_ (0.115 g, 0.194 mmol) was mixed with DMF (1 mL) and left to sit for 24 hours, covered. The resulting blue powder was then isolated by filtration and the composition of the final product was found to be Ni[Au(CN)_2_]_2_(DMF)_2_. Yield: 0.092 g, 69%. Anal. Calcd for C_10_H_14_N_6_Au_2_NiO_2_: C 17.09%, H 2.01%, N 11.96%. Found: C 16.95%, H 2.08%, N 12.24%. IR (KBr): 3,429(m), 2,994(w), 2,964(w), 2933(m), 2,892(w), 2,809(w), 2,189(s, ν_CN_), 1,658(s), 1,498(w), 1,434(m), 1,418(w), 1,384(m), 1,252(m), 1,109(s), 1,061(m), 689(s), 488(w) cm^−1^.

### 2.6. Synthesis of Co[Au(CN)_2_]_2_(Pyridine)_2_

Co(*μ*-OH_2_)_2_[Au(CN)_2_]_2_ (0.052 g, 0.088 mmol) was mixed with water/pyridine (2.5 mL, 95:5) and let to sit for 24 hours, covered. The powder became more orange, was isolated by filtration and dried in ambient air. The composition was found to be Co[Au(CN)_2_]_2_(pyridine)_2_. Yield: 0.049 g, 78%. Anal. Calcd for C_14_H_10_N_6_Au_2_Co: C 23.51%, H 1.41%, N 11.75%. Found: C 23.67%, H 1.55%, N 11.68%. IR (KBr): 3,086(w), 3,068(w), 3,053(w), 3,024(w), 2,168(s, ν_CN_), 1,605(s), 1,574(s), 1,486(m), 1,447(s), 1,242(w), 1,216(m), 1,157(w), 1,073(m), 1,041(m), 1,016(w), 946(w), 882(w), 759(m), 698(s), 633(m), 460(w), 431(m) cm^−1^.

### 2.7. Synthesis of Ni[Au(CN)_2_]_2_(Pyridine)_4_

A 96:4 water/pyridine solution (1.5 mL) of K[Au(CN)_2_] (0.059 g, 0.21 mmol) was prepared. This solution was added to a 96:4 water/pyridine solution (1 mL) of Ni(NO_3_)_2_·6H_2_O (0.030 g, 0.10 mmol). A purple powder was obtained immediately, which was filtered and air-dried. The product was determined to be Ni[Au(CN)_2_]_2_(pyridine)_4_. Yield: 0.070 g, 78%. Anal. Calcd for C_24_H_20_N_8_Au_2_Ni: C 33.02%, H 2.31%, N 12.83%. Found: C 32.64%, H 2.25%, N 12.55%. IR (KBr): 3,095(w), 3,070(w), 3,041(w), 3,023(w), 2,993(w), 2,924(w), 2,855(w), 2,171(s, ν_CN_), 2,143(s, ν_CN_), 1,602(s), 1,574(m), 1,488(m), 1,449(s), 1,443(s), 1,237(w), 1,216(m), 1,151(w), 1,068(m), 1,042(m), 1,011(w), 769(m), 756(m), 699(s), 631(m), 469(w), 435(m) cm^−1^.

### 2.8. Synthesis of Co[Au(CN)_2_]_2_

Co(μ-OH_2_)_2_[Au(CN)_2_]_2_ was heated to 145 °C in an oven for 2 hours to afford cobalt-blue coloured Co[Au(CN)_2_]_2_ quantitatively. The product is stable in air for approximately two days, and was stored under an inert atmosphere to prevent rehydration. Anal. calcd. for C_4_N_4_Au_2_Co: C, 8.63%; H, 0%; N, 10.06%. Found: C, 8.51%; H, trace%; N, 9.67%. IR (ATR): 2,177 (s, ν_CN_), 2,140 cm^−1^ (vw, ^13^ν_CN_).

### 2.9. Single Crystal X-Ray Structure of Zn[Au(CN)_2_]_2_(DMSO)_2_

A 0.42 × 0.22 × 0.14 mm^3^ crystal of Zn[Au(CN)_2_]_2_(DMSO)_2_ was mounted on a glass fiber using epoxy adhesive. The data were collected at room temperature on an Enraf Nonius Delft CAD-4 diffractometer with a PMT point detector using the control program DIFRAC [44]. A Mo K_α_ normal-focus sealed tube operated at 0.736 kW power (46 kV, 16 mA) was utilized for data collection over the range 4° < 2θ < 60°. The data was corrected by integration for the effects of absorption using a semi-empirical psi-scan method with a 0.073–0.262 transmission range. Data reduction included corrections for Lorentz and polarization effects using the NRCVAX software suite [45]. Final unit-cell dimensions were determined based on 43 well-centred reflections with a range of 47° < 2θ < 51°. The structure of Zn[Au(CN)_2_]_2_(DMSO)_2_ was solved in CRYSTALS [46] using direct methods (SIR92) and expanded using Fourier techniques. Diagrams were prepared using ORTEP-3 [47] and POV-Ray [48].

The coordinates and anisotropic temperature factors for all non-hydrogen atoms were refined. The final refinement using observed data (I_o_ ≥ 2.50 σ(I_o_)) included an extinction parameter, and statistical weights with 177 parameters for 2,953 unique reflections. Hydrogen atoms were placed in calculated positions (d_C-H_ 0.95 Å) and initially refined with soft restraints on the bond lengths and angles to regularise their geometry; afterwards their coordinate shifts were linked with those of the respective carbon atoms during refinement. Isotropic thermal parameters for the hydrogen atoms were initially assigned proportionately to the equivalent isotropic thermal parameters of their respective carbon atoms. Subsequently the isotropic thermal parameters for the H atoms on the DMSO were constrained to have identical shifts during refinement. Crystallographic data for Zn[Au(CN)_2_]_2_(DMSO)_2_ are tabulated in Table 2. Selected bond lengths and angles are given in Table 3.

### 2.10. Powder X-Ray Data Indexing/Structure Determination of M[Au(CN)_2_]_2_(analyte)_x_ (M = Co and Ni)

The structures of Ni[Au(CN)_2_]_2_(DMF)_2_ and Co[Au(CN)_2_]_2_(pyridine)_2_ were solved and refined using the DASH software package [49]. After indexing the experimental diffractogram, an automatic background fitting and subtraction was performed, followed by peak fitting to extract the intensity of each peak. To solve the crystal structure, a predefined building block was used. The asymmetric units of Co[Au(CN)_2_]_2_(DMF)_2_ [50] and Cu[Au(CN)_2_]_2_(pyridine)_2_ [35] were chosen as the building blocks used to solve the structure of Ni[Au(CN)_2_]_2_(DMF)_2_ and Co[Au(CN)_2_]_2_(pyridine)_2_ respectively. Optimization of the position and orientation of this building block was performed using a simulated annealing algorithm to maximize the agreement with the experimental diffractogram (relative intensity and peak position). The coordinates of each atom in the building blocks were further optimized once a structural model was obtained. The orientation of the pyridine molecules in Co[Au(CN)_2_]_2_(pyridine)_2_ was not refined as the diffractogram is mainly affected by the atomic position of the heavy atoms and slight changes in the position of the pyridine molecules do not cause noticeable changes in the diffractogram. The atomic coordinates for Ni[Au(CN)_2_]_2_(DMF)_2_ and Co[Au(CN)_2_]_2_(pyridine)_2_ are reported in Tables S1 and S2, respectively. Due to the poor quality of the powder diffractogram, the structure of Ni[Au(CN)_2_]_2_(DMSO)_2_ could not be determined using DASH. The diffractogram could, however, be indexed using WinPLOTR [51] to a unit cell very similar to that of the analogous Zn-containing polymer. The powder diffractogram for Co[Au(CN)_2_]_2_(DMSO)_2_ was also poor and was therefore only indexed with WinPLOTR but not utilized to generate an atomic resolution structure.

## 3. Results and Discussion

**Figure 1.**
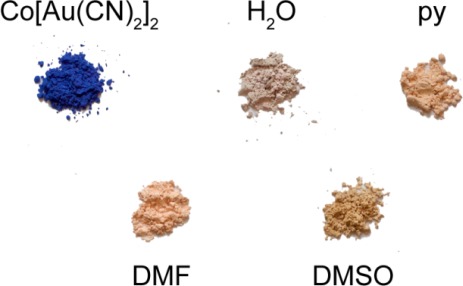
Powders of Co[Au(CN)_2_]_2_ (**top left**), Co(μ-OH_2_)_2_[Au(CN)_2_]_2_ (**top middle**), Co[Au(CN)_2_]_2_(pyridine)_4_ (**top right**), Co[Au(CN)_2_]_2_(DMF)_2_ (**bottom left**) and Co[Au(CN)_2_]_2_(DMSO)_2_ (**bottom right**). The latter three were synthesized by vapour absorption by solid Co[Au(CN)_2_]_2_.

The vapochromic behaviour of the M(*μ*-OH_2_)_2_[Au(CN)_2_]_2_ (M = Co, Ni) coordination polymers was investigated by exposing them to vapours of DMSO, DMF and pyridine for several hours/days in a sealed container, after which the FT-IR and UV-vis-NIR spectra and the powder X-ray diffractogram of the product were collected. The ν_CN_ bands for each product are reported in Table 1 and compared with those of the product made from solution (see below); visible colour changes were more noticeable for the Co(II) system (Figure 1) than for the Ni(II) analogue. Details for each metal/VOC vapour combination are outlined below.

### 3.1. DMSO-Containing Complexes

Upon exposure to DMSO, the pink Co(*μ*-OH_2_)_2_[Au(CN)_2_]_2_ coordination polymer (λ_max_ ≥ 1110, 525 nm) was converted over a three day period to a lighter, brighter pink coloured product (Figure 1; λ_max_ = 1,040, 500, 470 nm) with a ν_CN_ band at 2,176 cm^−1^ (Table 1). This material was stable for months after exposure to ambient atmospheric conditions, indicating that replacement of DMSO molecules for water molecules does not occur at room temperature, which contrasts with the two previously reported Cu[Au(CN)_2_]_2_(DMSO)_2_ polymorphs that readily undergo conversion when exposed to ambient moisture [35]. On the other hand, when Ni(*μ*-OH_2_)_2_[Au(CN)_2_]_2_ was exposed to DMSO vapour for one week, ν_CN_ bands attributable to the starting material were still present, as well as new bands at 2,181/2,164 cm^−1^. No visible colour change occurred.

Since VOC absorption by M(*μ*-OH_2_)_2_[Au(CN)_2_]_2_ powders does not lead to single crystals suitable for structural analysis, in order to assist in deciphering the identity and structure of these M[Au(CN)_2_]_2_(DMSO)_x_ products, the preparation of the analogous materials via solution methods was targeted. Thus, the reaction of Co(II), Ni(II) and Zn(II) salts with K[Au(CN)_2_] in DMSO was conducted; the Zn(II) analogue is included since it is the only one which forms single crystals from DMSO. The Ni and Zn reactions afforded the formation of a blue/turquoise precipitate (Ni) or colourless crystals (Zn) of M[Au(CN)_2_]_2_(DMSO)_2_, determined by elemental analysis. The FT-IR spectra show two strong bands in the cyanide stretching region (ν_CN_ = 2,189/2,180 and 2,186/2,175 cm^−1^ for the Ni and Zn systems, respectively) as well as bands attributable to the sulfoxide (S=O) moiety (1,032–956 cm^−1^) [52]. The slight shift in ν_CN_ between the Ni and Zn materials can be attributed to the different transition metal bound to the nitrogen atom of each cyanide group [53].

**Figure 2.**
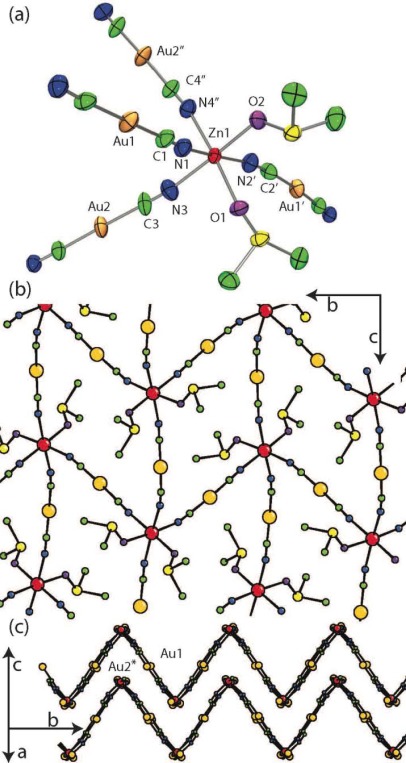
Extended 2-D structure of Zn[Au(CN)_2_]_2_(DMSO)_2_. (**a**) Local geometry of Zn, showing thermal ellipsoids; (**b**) A single 2-D sheet, viewed down the face of the sheet; (**c**) A pair of 2-D sheets, viewed perpendicular to the sheet face, long Au(1)-Au(2*) interactions 3.4943(6) Å represents the closest contact between sheets (DMSO molecules excluded for clarity).

The Zn[Au(CN)_2_]_2_(DMSO)_2_ structure (Figure 2, Tables 2 and 3) contains a single octahedral Zn(II) centre, with two *cis*-coordinated oxygen-bound DMSO molecules (Figure 2(a), Zn-O = 2.120(6) and 2.075(6) Å). The remaining four sites around the Zn(II) are coordinated with [Au(CN)_2_]^−^ units with an average Zn-N bond length of 2.144(8) Å, longer than the <2.0 Å Zn-N bond lengths in the tetrahedrally coordinated polymorphs of Zn[Au(CN)_2_]_2_ [37,38]. A 2-D corrugated structure, with DMSO molecules protruding from the apex of the corrugation, is formed by the bridging [Au(CN)_2_]^−^ units (Figure 2(c)). The closest contact between sheets is a Au-Au interaction of 3.4943(6) Å. A similar crystal structure was observed for the Cu(II) system [35].

**Figure 3.**
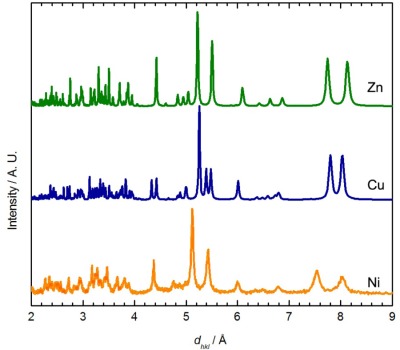
Comparison between the powder X-ray diffractograms predicted for Zn[Au(CN)_2_]_2_(DMSO)_2_ (Zn, green) and the blue Cu[Au(CN)_2_]_2_(DMSO)_2_ polymorph (Cu, blue), with the diffractogram obtained experimentally for Ni[Au(CN)_2_]_2_(DMSO)_2_ (Ni, orange), prepared via solution methods.

Although no single crystal of Ni[Au(CN)_2_]_2_(DMSO)_2_ suitable for X-ray crystallography could be obtained, its powder X-ray diffractogram showed similar features to the diffractograms of the Zn[Au(CN)_2_]_2_(DMSO)_2_ and Cu[Au(CN)_2_]_2_(DMSO)_2_ (blue polymorph) coordination polymers (Figure 3) [35]. The diffractogram of Ni[Au(CN)_2_]_2_(DMSO)_2_ could be indexed to a monoclinic unit cell, with parameters very similar to those of the Zn analogue (Table 4). Thus, the structure of Ni[Au(CN)_2_]_2_(DMSO)_2_ likely consists of octahedral Ni(II) ions coordinated by two DMSO molecules in a *cis* fashion and connected to four other Ni(II) centres via [Au(CN)_2_]^−^ units, generating corrugated 2-D sheets; due to the poor quality of the diffractogram, no effort was made to structurally model the data any further. Note that the ν_CN_ data and powder X-ray diffractograms for Ni[Au(CN)_2_]_2_(DMSO)_2_ do not perfectly match those obtained via vapour absorption of DMSO by Ni(*μ*-OH_2_)_2_[Au(CN)_2_]_2_, indicating that a related, but different structure is likely accessed by vapour absorption.

Although the analogous reaction between a Co(II) salt and K[Au(CN)_2_] in neat DMSO yielded an inseparable mixture of products, when pink Co(*μ*-OH_2_)_2_[Au(CN)_2_]_2_ was immersed in DMSO for three days, a slight colour change occurred. The resulting light pink powder was isolated and the chemical composition was determined to be Co[Au(CN)_2_]_2_(DMSO)_2_. The FT-IR spectrum of this product contains only one ν_CN_ band (2,176 cm^−1^), analogous to that observed by vapour absorption (Table 1).

**Figure 4.**
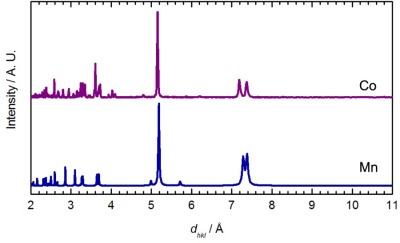
Comparison between the powder X-ray diffractogram determined experimentally for the Co[Au(CN)_2_]_2_(DMSO)_2_ (Co, purple) and the diffractogram predicted for Mn[Au(CN)_2_]_2_(H_2_O)_2_ from the single crystal structure (Mn, blue) [54].

This reaction did not yield single crystals suitable for X-ray analysis, only microcrystalline powder. The powder diffractogram (Figure 4) was indexed and the unit cell parameters obtained are reported in Table 4. The diffractogram and the unit cell parameters do not match that of the Zn, Cu or Ni-containing M[Au(CN)_2_]_2_(DMSO)_2_ polymers (Figure 3 and Table 4), which suggests that a different structural arrangement is adopted. Due to the poor quality of the diffractogram, no structure refinement could be performed, but a structural model is proposed based on comparisons with other known complexes.

The presence of only one ν_CN_ band, shifted from free [Au(CN)_2_]^−^, suggests that all the cyanide groups are in a similar environment and N-bound to Co(II). This could be the case if the structure of Co[Au(CN)_2_]_2_(DMSO)_2_ contained square-grid arrays of Co[Au(CN)_2_]_2_ with DMSO molecules bound to Co(II) ions in a *trans* fashion, on each side of the grid. This type of structure has been observed for the previously reported Co[Au(CN)_2_]_2_(DMF)_2_ [50] and Mn[Au(CN)_2_]_2_(H_2_O)_2_ [54] coordination polymers. Although the ν_CN_ band observed for Co[Au(CN)_2_]_2_(DMSO)_2_ (2,176 cm^−1^) is similar to that for the DMF analogue (2,179 cm^−1^) [35], the powder diffractograms are a poor match.

The main features in the diffractograms of Co[Au(CN)_2_]_2_(DMSO)_2_ and Mn[Au(CN)_2_]_2_(H_2_O)_2_ [54] are similar (Figure 4). The packing in Co[Au(CN)_2_]_2_(DMF)_2_ differs from Mn[Au(CN)_2_]_2_(H_2_O)_2_ since the Co[Au(CN)_2_]_2_ layers stack in discrete pairs whereas the Mn[Au(CN)_2_]_2_ layers are equally spaced and do not form pairs of layers. It is hence suggested that, analogous to the Mn[Au(CN)_2_]_2_(H_2_O)_2_ superstructure, the Co[Au(CN)_2_]_2_(DMSO)_2_ polymer contains flat and equally spaced square-grid arrays, with DMSO molecules bound in a *trans* fashion.

With this information in hand, it is clear that vapour absorption of DMSO by Co(*μ*-OH_2_)_2_[Au(CN)_2_]_2_ gives Co[Au(CN)_2_]_2_(DMSO)_2_ cleanly; the solution method yields a product with comparable UV-vis, powder X-ray and IR data. However, the Ni(II) system does not run to completion, even after a week of vapour exposure, and the products from vapour and solution methods, although related, do not appear to be identical.

### 3.2. DMF-Containing Complexes

**Figure 5.**
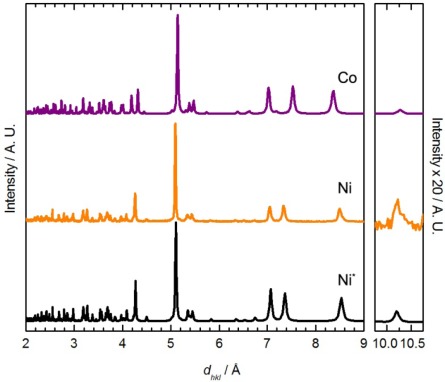
Comparison between the powder X-ray diffractogram (generated from single-crystal data) for Co[Au(CN)_2_]_2_(DMF)_2_ (Co, purple) [50], the experimental diffractogram for Ni[Au(CN)_2_]_2_(DMF)_2_ (Ni, orange) and the diffractogram predicted by the proposed structural model (Ni*, black).

When Co(*μ*-OH_2_)_2_[Au(CN)_2_]_2_ was exposed to DMF vapour, faint colour changes to a light, bright pink (λ_max_ = 1035, 490, 468 nm) were observed after several hours (Figure 1), and the IR spectrum indicated a clean conversion; a single ν_CN_ peak at 2,182 cm^−1^ was generated (Table 1). The analogous solution-based synthetic product has a comparable FTIR spectrum and powder X-ray diffractogram to that obtained by vapour absorption, both of which are indistinguishable from that of the previously published Co[Au(CN)_2_]_2_(DMF)_2_ system (Figure 5) [50]; this is therefore the product that is obtained. For the Ni(II) analogue, after one week of being exposed to DMF vapour, the FT-IR spectrum of the product showed the presence of the starting Ni(*μ*-OH_2_)_2_[Au(CN)_2_]_2_ compound as well as an additional band at 2,184 cm^−1^; no significant colour change was observed.

For comparison, when Ni(*μ*-OH_2_)_2_[Au(CN)_2_]_2_ was immersed in DMF for one day, a colour change from light green to blue was observed, similar to Co[Au(CN)_2_]_2_(DMF)_2_. The FT-IR spectrum of the isolated product showed only one ν_CN_ band at 2,189 cm^−1^. The chemical composition was determined to be Ni[Au(CN)_2_]_2_(DMF)_2_ from elemental analysis, comparable to the previously reported Co[Au(CN)_2_]_2_(DMF)_2_ coordination polymer [50] but different from that of the Cu(II)-containing analogue, in which only one DMF molecule was incorporated. The unit cell obtained from indexing the powder X-ray diffractogram of Ni[Au(CN)_2_]_2_(DMF)_2_ (Figure 5-Ni) is also similar to that of Co[Au(CN)_2_]_2_(DMF)_2_ (Table 5) [50].

**Figure 6.**
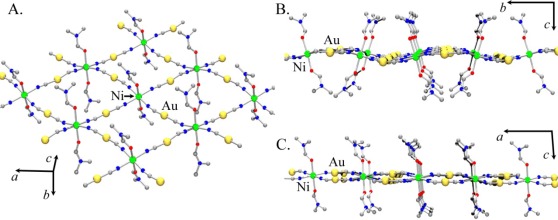
Structural model proposed for Ni[Au(CN)_2_]_2_(DMF)_2_: (**A**). A 2-D square grid array with DMF molecules coordinated on both sides of the Ni[Au(CN)_2_]_2_ layer; (**B**) and (**C**). Side-view of a layer showing the relative position of the [Au(CN)_2_]^−^ units with respect to the Ni(II) centres.

Using the atomic coordinates of the published Co(II) analogue as a starting point, the structure of Ni[Au(CN)_2_]_2_(DMF)_2_ was determined from the observed powder X-ray diffractogram (the atomic coordinates are reported in Table S1). Figure 6 shows the proposed model for Ni[Au(CN)_2_]_2_(DMF)_2_.

As with Co[Au(CN)_2_]_2_(DMF)_2_, the structure contains 2-D square grids of Ni[Au(CN)_2_]_2_ (Figure 6(A)). The DMF molecules are O-bound to the Ni(II) centres on each side of the grids, which stack in an offset manner to accommodate the DMF molecules. The [Au(CN)_2_]^−^ units are slightly bent and sit above and below the plane containing the Ni(II) centres (Figure 6(B,C)). As observed in the Co[Au(CN)_2_]_2_(DMF)_2_ structure, the layers stack in discrete pairs with aurophilic interactions of *ca.* 3.3 Å forming the pair, whereas Au-Au distances of *ca.* 5.4 Å separate each pair of layers.

Thus, both Co(II) and Ni(II) analogues of M(*μ*-OH_2_)_2_[Au(CN)_2_]_2_ sense DMF as an analyte, although the Co(II) system reacts more cleanly and quickly. Both form similar 2-D layer structures of M[Au(CN)_2_]_2_(DMF)_2_, with the same product being generated from solution or vapour absorption. However, the Ni(II) analogue only partially converts to product in the vapour absorption experiment, and as a result the expected visible vapochromism (green to blue by solution methods) was not observed; this putative vapochromic response was likely attenuated by the residual absorption bands of the unreacted starting material.

### 3.3. Pyridine-Containing Complexes

When Co(*μ*-OH_2_)_2_[Au(CN)_2_]_2_ was exposed to pyridine vapour, conversion to a light pink product (λ_max_ = 1,000, 525, 485 nm, Figure 1) having ν_CN_ bands at 2,168/2,144 cm^−1^ occurred (Table 1). Upon exposure to pyridine vapour, Ni(*μ*-OH_2_)_2_[Au(CN)_2_]_2_ undergoes a colour change, from light green to blue over several days. The FT-IR spectrum shows ν_CN_ bands of the starting material and additional bands at 2,170/2,162/2,141 cm^−1^; this complexity likely indicates the formation of a mixture of analyte-containing products.

On the solution synthesis side, when a powdered sample of Co(*μ*-OH_2_)_2_[Au(CN)_2_]_2_ was immersed for one day in a mixture of water and pyridine (96:4), a slight colour change was observed. The FT-IR spectrum of the isolated powder showed only one strong ν_CN_ band at 2,168 cm^−1^ (Table 1), reminiscent of that of the Co[Au(CN)_2_]_2_(DMF)_2_ system. Thus, a different product is obtained in solution. Pyridine-based bands could also be observed.

**Figure 7.**
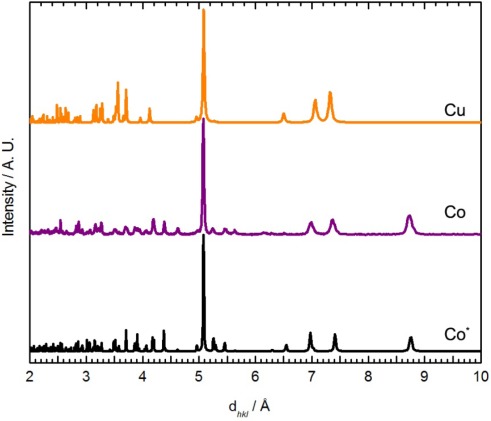
Comparison between the powder X-ray diffractogram (generated from single-crystal data) for Cu[Au(CN)_2_]_2_(pyridine)_2_ (Cu, orange) [35], the experimental diffractogram of Co[Au(CN)_2_]_2_(pyridine)_2_ (Co, purple), and the diffractogram predicted by its structural model (Co*, black).

The powder X-ray diffractogram of the solution-based product, which analyzes as Co[Au(CN)_2_]_2_(pyridine)_2_, is shown in Figure 7 and is different than the material obtained by vapour diffusion; it also shows similar features to the diffractogram of Co[Au(CN)_2_]_2_(DMF)_2_ (Figure 5) and Cu[Au(CN)_2_]_2_(pyridine)_2_. The diffractogram was indexed and the optimized unit cell parameters are reported in Table 5. They are comparable with those of the Ni and Co-containing M[Au(CN)_2_]_2_(DMF)_2_ complexes (Figure 6).

**Figure 8.**
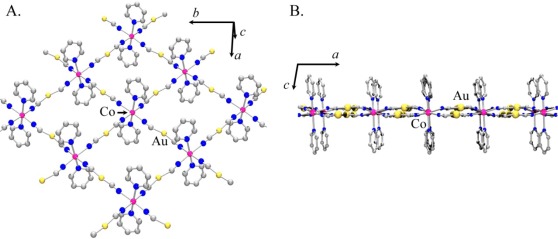
Structural model proposed for Co[Au(CN)_2_]_2_(pyridine)_2_: (**A**) 2-D square-grid array with pyridine molecules on both sides of the grid; (**B**) Side-view of a 2-D layer, showing the position of the [Au(CN)_2_]^−^ units with respect to the Co(II) centres.

Using a combination of the Cu[Au(CN)_2_]_2_(pyridine)_2_ and Co[Au(CN)_2_]_2_(DMF)_2_ structures as a starting point, a structural model is proposed for Co[Au(CN)_2_]_2_(pyridine)_2_ (the corresponding atomic coordinates are reported in Table S2). The structure consists of 2-D square grids of Co[Au(CN)_2_]_2_ with pyridine molecules N-bound to the Co(II) centres on both sides of the layer (Figure 8(A)). The structure differs from the Cu(II) analogue as all the cyanide groups are equidistant from the Co(II) centres and no Jahn-Teller distortion is observed. Also, the Co[Au(CN)_2_]_2_ layers are not completely flat as in the Cu[Au(CN)_2_]_2_ system, but the [Au(CN)_2_]^−^ units are buckled in an alternate way on each side of the layer (Figure 8(B)), as is observed in the related Co[Au(CN)_2_]_2_(DMF)_2_ polymer [50]. This arrangement allows π–π interactions between the pyridine rings, but no aurophilic interactions are present as the closest contact between two Au atoms is approx. 3.7 Å. The powder diffractogram predicted by this model is compared to the experimental diffractogram of Co[Au(CN)_2_]_2_(pyridine)_2_ in Figure 7.

The addition of Ni(NO_3_)_2_·6H_2_O to KAu(CN)_2_ in a mixture of water and pyridine (96:4) generated purple Ni[Au(CN)_2_]_2_(pyridine)_4_, confirmed by elemental analysis. The FT-IR spectrum of this product shows two ν_CN_ bands (2,171 and 2,143 cm^−1^), consistent with two of the three bands observed by vapour diffusion. Depending on the reaction conditions, variable amounts of an uncharacterized material could also be generated (as indicated by IR: 2182, 2153 cm^−1^) but a pure sample could not be isolated.

Clearly, pyridine as an analyte yields the most complex, least clean conversions for both Co(II) and Ni(II) analogues. Thus, for Co(II), a bis-pyridine adduct forms from aqueous pyridine solution, while vapour absorption methods generated a different product. A Ni[Au(CN)_2_]_2_(pyridine)_4_ complex was isolated via solution methods, confirmed by elemental analysis. The powder X-ray diffractograms of Ni[Au(CN)_2_]_2_(pyridine)_4_ and the Co vapochromic product are very similar, which suggests that the product of pyridine vapour with the Co(II) system is a tetrakis-pyridine adduct. Finally, pyridine vapour absorption by Ni(*μ*-OH_2_)_2_[Au(CN)_2_]_2_ is the least clean of all the reactions studied herein, generating Ni[Au(CN)_2_]_2_(pyridine)_4_, another unidentified product (perhaps a bis-pyridine adduct) as well as leaving residual starting material.

### 3.4. Analyte-Free M[Au(CN)_2_]_2_ Systems

Analyte-free systems were investigated in order to see if the vapochromic response would be improved without the presence of an initial analyte molecule (H_2_O) in the structure. Co[Au(CN)_2_]_2_ was synthesized by heating Co(μ-OH_2_)_2_[Au(CN)_2_]_2_ to at least 110 °C for a minimum of one hour (ideally, 145 °C for two hours; heating at 180 °C for 10 minutes was very recently reported as an alternative synthesis [42]). The compound changes from a light pink colour (octahedral Co(II)) to an intense cobalt-blue colour (tetrahedral Co(II)). Co[Au(CN)_2_]_2_ is stable in ambient air for approximately two days, whereupon it rehydrates to a compound with a similar powder X-ray diffractogram and IR spectrum to Co(μ-OH_2_)_2_[Au(CN)_2_]_2_, but is a light purple colour; its identity is under investigation.

**Figure 9.**
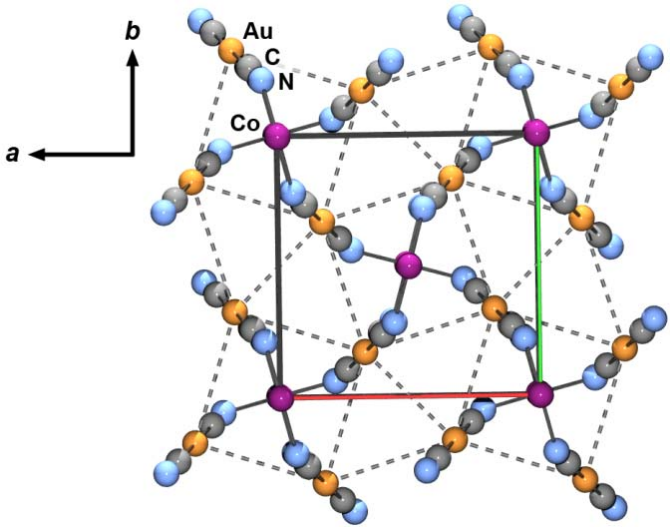
Extended 3-D structure of Co[Au(CN)_2_]_2_, looking down the *c* axis. Dashed lines represent Au-Au interactions.

The structure of Co[Au(CN)_2_]_2_ was deduced by matching the powder X-ray diffractogram to that of the isostructural compound [37], γ-Zn[Au(CN)_2_]_2_ (Figure S1), indexing the data accordingly (spacegroup *P*-4*b*2*, a* = 6.7968(17) Å, *c* = 8.4651(3) Å) and refining atomic positions (Tables S3 and S4). The cobalt(II) centres are tetrahedrally coordinated through the nitrogen end of four [Au(CN)_2_]^−^ units, creating a tetragonal diamond-type lattice (Figure 9). There are four interpenetrated networks that are linked together via aurophilic interactions (3.33 and 3.56 Å). Note that a previously reported synthesis of Co[Au(CN)_2_]_2_ (by a simple addition of salt [55]) generated a different polymorph (equivalent to quartz-type α-Zn[Au(CN)_2_]_2_) [37,38], while using another route, via solvent-free grinding of Co(OH_2_)_2_[Au(CN)_2_]_2_, the structure was not determined [42].

Vapochromic experiments were also performed on Co[Au(CN)_2_]_2_ with DMF, DMSO, and pyridine as analytes. Uptake occurred in all cases, yielding Co[Au(CN)_2_]_2_(analyte)_x_ materials in similar shades of light pink; with DMF as the analyte, the complex showed the fastest conversion (about 1 day), whereas pyridine showed the slowest conversion (several days). Based on IR and powder X-ray diffraction data, the complexes that form using DMF and DMSO are identical to those produced from vapour absorption by Co(μ-OH_2_)_2_[Au(CN)_2_]_2_. The complex with pyridine shows two ν_CN_ bands at 2,170 and 2,142 cm^−1^ and a powder X-ray diffractogram matching the Co[Au(CN)_2_]_2_(pyridine)_4_ adduct identified via vapour absorption by Co(μ-OH_2_)_2_[Au(CN)_2_]_2_, rather than the bis-pyridine adduct obtained by solution methods. These IR spectroscopic results broadly match those very recently reported for the addition of a range of donor solvents (including pyridine, DMSO and DMF, as reported here) to Co[Au(CN)_2_]_2_ [42].

The synthesis of Ni[Au(CN)_2_]_2_ was attempted by several methods but the desired product could not be obtained. Since the dehydration temperature for Ni(μ-OH_2_)_2_[Au(CN)_2_]_2_ is between 215–260 °C, it is necessary to heat above 260 °C for extended periods of time to dehydrate it, but doing so resulted in decomposition. Attempts to effect chemical dehydration by stirring Ni(μ-OH_2_)_2_[Au(CN)_2_]_2_ in glacial acetic acid or refluxing in trimethylsilylchloride resulted in no change. The addition of [^n^Bu_4_N][Au(CN)_2_] [56] to anhydrous NiCl_2_·DME in dry organic solvents under an inert, dry atmosphere yielded other products which are under investigation [57], but did not generate Ni[Au(CN)_2_]_2_. It therefore appears that Ni(μ-OH_2_)_2_[Au(CN)_2_]_2_ is exceptionally stable, which is in agreement with the vapour absorption results, which indicate only partial conversion in every case.

### 3.5. Comparison of M[Au(CN)_2_]_2_-Based Vapochromic Materials (M = Co, Ni, Cu)

The M[Au(CN)_2_]_2_(analyte)_x_ polymers show visible vapochromism since each analyte molecule that is incorporated binds to the M(II) centre and modifies differently its crystal field splitting. As a consequence, the colours of the vapochromic compounds change as the *d–d* absorbance bands shift [35,36]. Table 6 shows the solid-state UV-vis-NIR absorbance maxima for the analyte-containing compounds prepared from solution (*vs.* direct analyte absorption by the solid).

The data in Table 6 and Figure 1 suggests that identification of the different M[Au(CN)_2_]_2_(analyte)_2_ polymers (M = Co, Ni) purely on the basis of colour cannot be accomplished as easily as for the Cu(II)-based analogues [35,36]. The M(II) coordination sphere in the M[Au(CN)_2_]_2_(analyte)_2_ polymers is very similar: an octahedral geometry with four cyanide groups occupying the same plane. The only difference lies in the two donor analytes that are *trans* to each other (M(NC)_4_(O-ligand)_2_ *vs.* M(NC)_4_(N-ligand)_2_). As a result, the crystal field splitting of the M(II) centres in each M[Au(CN)_2_]_2_(analyte)_2_ is very similar and, hence, so is their colour, particularly for DMSO and DMF analytes; some differentiation for pyridine is possible. The spectra of the M(*μ*-OH_2_)_2_[Au(CN)_2_]_2_ polymers containing M(NC)_2_(O-ligand)_4_ centres is, however, shifted to slightly lower energy. The analyte-free Co[Au(CN)_2_]_2_ system is also markedly different from all Co(II)-analyte adducts, consistent with tetrahedral *vs.* octahedral Co(II) centres.

Upon comparing the reactivity of the M[Au(CN)_2_]_2_-based systems, analyte exchange is quickest for M = Cu(II) and Zn(II) [35–38], followed by the Co(II) system and least so in the Ni(II) system, for which analyte exchange was found to be only partial, with a mixture of products obtained; this is likely due to the relative kinetic inertness of Ni(II) complexes. Thus, the Ni(II)-systems are not likely to be very useful vapochromic materials for VOC sensing. Indeed, the most easily visible vapochromic system reported here is the analyte-free Co[Au(CN)_2_]_2_, for which drastic colour changes upon analyte-binding occurs, but differentiation of the products obtained with different analytes is more difficult. Note that other factors, such as particle size/surface area and analyte vapour pressure, also influence the overall kinetic response of the material.

**Scheme 1.**
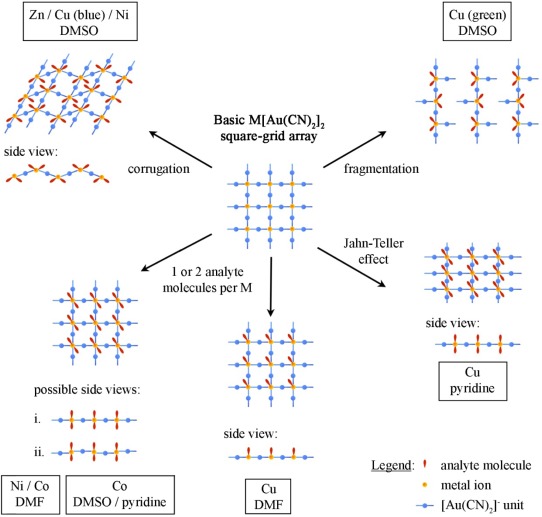
Different structural models observed for the M[Au(CN)_2_]_2_(analyte)_x_ polymers, all resulting from the structural flexibility of the basic M[Au(CN)_2_]_2_ square-grid array.

From a structural perspective, it is known that a system does not need to be porous in order to undergo guest uptake [58]. For example, a flexible metal-ligand superstructure can dynamically adapt in order to accommodate a variety of potential guests, as is the case here [58–64]. Despite the range of analyte molecules utilized, the basic structural motif of the M[Au(CN)_2_]_2_(analyte)_x_ coordination polymers remained the same in most cases: a 2-D square-grid M[Au(CN)_2_]_2_ network was the basic structural feature, which is flexible and can adapt to accommodate the requisite structural changes, depending on the metal ion and analyte type. Scheme 1 summarizes the different types of structural distortions observed for the M[Au(CN)_2_]_2_-based systems (M = Co, Ni, Cu).

The major mode of flexibility [62–64] lies in the fact that the 2-D square-grid can be entirely flat, as in the M[Au(CN)_2_]_2_(DMF)_2_ or M[Au(CN)_2_]_2_(pyridine)_2_ complexes, or it can buckle to generate a corrugated 2-D array, as observed in several M[Au(CN)_2_]_2_(DMSO)_2_ (M = Zn, Ni or Cu (blue polymorph)) polymers. In addition, in a flat square-grid, the [Au(CN)_2_]^−^ units can lie completely co-planar with the M(II) centres as in Cu[Au(CN)_2_]_2_(pyridine)_2_ or bent out of the M(II)-containing plane, as in M[Au(CN)_2_]_2_(DMF)_2_. For a given analyte, similar structures are usually observed despite the change in metal centre. DMSO is the only type of guest utilized that forces corrugation of the layers, whereas DMF and pyridine favour flat square-grid arrays to be obtained.

Due to Jahn-Teller distortions, the structures of the polymers containing Cu(II) ions are more axially elongated than that of the Ni(II) and Co(II)-containing polymers. This form of structural flexibility is particularly important since substantially different FT-IR signatures in the cyanide vibration region for the Cu(II)-analogues are generated depending on the axial/equatorial cyanide bonding arrangement in the system; this detection methodology is less striking for the Co/Ni analogues, which don’t have the same geometric flexibility as Cu(II) [35]. In the Cu[Au(CN)_2_]_2_(analyte)_2_ system, the differences in cyanide vibration frequency between certain analyte adducts vary by up to 70 cm^−1^. However, the differentiation between DMF, DMSO, and pyridine analytes in the Co(II) analogue is poorer due to similar colour and ν_CN_ frequencies. The FT-IR signatures in the cyanide vibration region for the Ni[Au(CN)_2_]_2_(analyte)_x_ polymers, on the other hand, are different from each other, allowing for analyte identification if analyte exchange actually occurs.

## 4. Conclusions

In summary, the Co[Au(CN)_2_]_2_-based systems react when exposed to different analytes, but detection and identification of the analyte by FT-IR and UV-vis spectroscopies are more difficult than in the analogous Cu(II) system, except for the analyte-free Co[Au(CN)_2_]_2_, which undergoes large structural and visible colour changes upon analyte-binding, albeit slowly. The Ni(II)-based system is relatively inert and does not undergo analyte exchange easily, at least with the analytes tested in this study, although the identity of the analyte present is more easily discerned than for the analogous Co(II) system. Nevertheless, it is clear that a flexible non-porous M[Au(CN)_2_]_2_-superstructure can readily adapt to integrate selected analytes, the efficacy and spectroscopic signatures of which depend on the metal cation in question. An examination of new M[Au(CN)_2_]-based systems with respect to detecting potential VOC analytes with different donor characteristics is worthwhile and efforts are underway towards this end.

## Supplementary Information

Unit cell parameters and powder X-ray diffractogram for Co[Au(CN)_2_]_2_, atomic coordinates for the powder X-ray diffraction models for Ni[Au(CN)_2_]_2_(DMF)_2_, Co[Au(CN)_2_]_2_(pyridine)_2_, and Co[Au(CN)_2_]_2_, and X-ray crystallographic data in cif format (CCDC Deposition #745836) for Zn[Au(CN)_2_]_2_(DMSO)_2_.



## Figures and Tables

**Table 1.** Cyanide vibration frequencies (ν_CN_, cm^−1^) for different M[Au(CN)_2_]_2_(analyte)_x_ (M = Co, Ni) complexes synthesized from solution and from vapour absorption.

**From Solution**		**From Absorption *^a^***	

**Complex**	**ν_CN_ (cm^−1^)**	**Vapour**	**ν_CN_ (cm^−1^)**
Co(μ-OH_2_)_2_[Au(CN)_2_]_2_	2,204(s), 2,168(s)		
Co[Au(CN)_2_]_2_(DMSO)_2_	2,177(s)	DMSO	2,176(s)
Co[Au(CN)_2_]_2_(DMF)_2_	2,182(s)	DMF	2,182(s)
Co[Au(CN)_2_]_2_(pyridine)_x_*^b^*	2,168(s)	pyridine	2,168(s), 2,144(s)
Ni(μ-OH_2_)_2_[Au(CN)_2_]_2_	2,214(s), 2,204(sh), 2,170(s)		
Ni[Au(CN)_2_]_2_(DMSO)_2_	2,189(s), 2,180(s)	DMSO	2,214(m), 2,203(w), 2,181(s), 2,170(s), 2,164(s)
Ni[Au(CN)_2_]_2_(DMF)_2_	2,189(s)	DMF	2,215(m), 2,189(s), 2,171(m)
Ni[Au(CN)_2_]_2_(pyridine)_4_	2,171(s), 2,143(s)	Pyridine	2,213(s), 2,202(m), 2,170(s), 2,162(s), 2,141(s)

a Solvent adducts were prepared from Co(μ-OH_2_)_2_[Au(CN)_2_]_2_ and from Ni(μ-OH_2_)_2_[Au(CN)_2_]_2_.

b x = 2 from solution and 4 from absorption methods.

**Table 2.** Crystallographic data for Zn[Au(CN)_2_]_2_(DMSO)_2_.

Formula	C_8_H_12_N_4_Au_2_O_2_S_2_Zn
Formula weight, *M_r_*	719.66
Colour	colourless
Shape	chunk
Dimensions, mm^3^	0.14 × 0.22 × 0.42
Crystal system	monoclinic
Space group	*P*2_1_/*c*
*Z*	4
*T*, K	293
*a*, Å	7.8403(11)
*b*, Å	12.8395(12)
*c*, Å	16.4572(17)
β, deg	98.891(10)
Volume, Å^3^	1636.8(3)
*D_calcd_*, g/cm^3^	2.920
*λ*, nm (Mo K_α_)	0.71073
2*θ* range, deg	4–60
*μ*, mm^−1^	19.592
Reflns, total	5010
Reflns, unique	4801
Reflns, *I_o_* ≥ 2.50 σ(*I_o_*)	2953
Extinction coefficient	16.0(15)
*R* (*F_o_*)	0.0342
*wR* (*F_o_*)	0.0396

**Table 3.** Selected bond lengths (Å) and angles (deg) for Zn[Au(CN)_2_]_2_(DMSO)_2_ *^a^*.

Zn(1)–N(1)	2.163(7)	Zn(1)–N(2′)	2.167(8)
Zn(1)–N(3)	2.140(8)	Zn(1)–N(4″)	2.104(8)
Zn(1)–O(1)	2.120(6)	Zn(1)–O(2)	2.075(6)
Au(1)–Au(2*)	3.4943(6)		
N(2′)–Zn(1)–N(4″)	91.3(3)	N(2′)–Zn(1)–O(1)	88.8(3)
N(4″)–Zn(1)–O(1)	175.7(3)	N(2′)–Zn(1)–O(2)	88.3(3)
N(4″)–Zn(1)–O(2)	91.2(3)	O(1)–Zn(1)–O(2)	93.2(3)
N(2′)–Zn(1)–N(1)	172.5(3)	N(4″)–Zn(1)–N(1)	93.0(3)
O(1)–Zn(1)–N(1)	87.4(3)	O(2)–Zn(1)–N(1)	85.4(3)
N(2′)–Zn(1)–N(3)	94.1(3)	N(4″)–Zn(1)–N(3)	87.9(3)
O(1)–Zn(1)–N(3)	87.8(3)	O(2)–Zn(1)–N(3)	177.4(3)
N(1)–Zn(1)–N(3)	92.3(3)	Zn(1)–O(1)–S(1)	126.3(4)
Zn(1)–O(2)–S(2)	126.0(4)	Zn(1)–N(1)–C(1)	172.8(8)
Zn(1′)–N(2′)–C(2′)	169.4(8)	Zn(1)–N(3)–C(3)	172.8(10)
Zn(1)–N(4″)–C(4″)	167.3(8)	Au(2*)–Au(1)–C(1)	105.3(3)

a Symmetry operations: ′ = x − 1, −y + 3/2, z − ½; ″ = −x + 4, y − 1/2, −z + 1/2;

* = −x + 4, −y + 2, −z + 1.

**Table 4.** Unit cell parameters determined for the M[Au(CN)_2_]_2_(DMSO)_2_ coordination polymers (M = Zn, Cu (blue polymorph), Ni and Co), by either single crystal or powder X-ray diffraction.

	**Zn**	**Cu (Blue)**	**Ni**	**Co**

**Sample**	**Crystal**	**Crystal**	**Powder**	**Powder**
crystal system	monoclinic	triclinic	monoclinic	monoclinic
space group	P21/c	P-1	P2_1_/c	C2/m
*a*, Å	7.8403(11)	7.874(7)	7.63	6.35
*b*, Å	12.8395(12)	12.761(11)	12.84	14.80
*c*, Å	16.4572(17)	16.207(13)	16.30	7.35
α, deg	90.0	89.61(7)	90.0	90.0
β, deg	98.891(10)	82.29(7)	98.89	100.77
γ, deg	90.0	88.57(7)	90.0	90.0
Volume, Å^3^	1,636.77	1,613.2(24)	1,577.65	677.52

**Table 5.** Unit cell parameters determined for the M[Au(CN)_2_]_2_(DMF)_2_ (M = Co, Ni) and Co[Au(CN)_2_]_2_(pyridine)_2_ coordination polymers by either single crystal or powder X-ray diffraction.

	**Co[Au(CN)_2_]_2_(DMF)_2_**	**Ni[Au(CN)_2_]_2_(DMF)_2_**	**Co[Au(CN)_2_]_2_(pyridine)_2_**

**Sample**	**Crystal^32^**	**Powder**	**Powder**
crystal system	monoclinic	monoclinic	monoclinic
space group	P2_1_/c	P2_1_/c	P21/c
*a*, Å	8.375(2)	8.5659	8.9334
*b*, Å	14.054(5)	14.1445	14.0712
*c*, Å	15.077(4)	14.7907	15.0425
α, deg	90.0	90.0	90.0
β, deg	92.75(2)	95.011	99.2538
γ, deg	90.0	90.0	90.0
Volume, Å^3^	1,772.6(9)	1785.2	1,866.3

**Table 6.** Absorbance maxima (λ_max abs_, nm) observed in the solid-state UV-Vis-NIR absorbance spectra of the M[Au(CN)_2_]_2_(analyte)_x_ (M = Co, Ni) coordination polymers prepared from solution.

**Ni(II) complexes**	**λ_max abs_ (nm)**		
Ni(*μ*-OH_2_)_2_[Au(CN)_2_]_2_	930	625	390
Ni[Au(CN)_2_]_2_(DMSO)_2_	925	620	385
Ni[Au(CN)_2_]_2_(DMF)_2_	925	610	385
Ni[Au(CN)_2_]_2_(pyridine)_4_	905	575	375

**Co(II) complexes**			

Co(*μ*-OH_2_)_2_[Au(CN)_2_]_2_	>1,110	525	
Co[Au(CN)_2_]_2_(DMSO)_2_	1,040	500	470
Co[Au(CN)_2_]_2_(DMF)_2_	1,035	490	468
Co[Au(CN)_2_]_2_(pyridine)_2_	1,000	525(sh)	485
Co[Au(CN)_2_]_2_	590	550 (sh)	365
