# Supplementary material for: Vapochromic Behaviour of M[Au(CN)2]2-Based Coordination Polymers (M = Co, Ni)
Source: Sensors (Basel). 2012 Mar 16;12(3):3669–92. doi: 10.3390/s120303669 (PMC3376558; doi:10.3390/s120303669)
Supplement: Supplementary file 1 [file sensors-12-03669-s001.pdf]

Supplementary Information

Vapochromic Behaviour of $M[Au(CN)_2]_2$-Based Coordination Polymers (M = Co, Ni)

Julie Lefebvre, Jasmine L. Korčok, Michael J. Katz and Daniel B. Leznoff *

Department of Chemistry, Simon Fraser University, 8888 University Drive, Burnaby, BC V5A 1S6, Canada; E-Mails: jlefebvr@ucalgary.ca (J.L.); jkorcok@tru.ca (J.L.K.); mjkatz@sfu.ca (M.J.K.)

* Author to whom correspondence should be addressed; E-Mail: dleznoff@sfu.ca;
Tel.: +1-778-782-4887; Fax: +1-778-782-3765.

Received: 10 February 2012; in revised form: 9 March 2012 / Accepted: 13 March 2012 / Published: 16 March 2012

Table S1. Fractional atomic coordinates for $Ni[Au(CN)_2]_2(DMF)_2$.

Atom	x	y	z	Occupancy
Au(1)	0.2354	0.2259	−0.0290	1
Au(2)	0.1372	−0.2393	0.5052	1
Ni(1)	0.1894	−0.0059	0.2379	1
O(1)	−0.0446	0.0332	0.2094	1
O(2)	0.4213	−0.0527	0.2608	1
N(1)	0.2384	0.0807	0.1293	1
N(2)	0.1418	−0.0901	0.3506	1
N(3)	0.2324	0.1077	0.3269	1
N(4)	0.1418	−0.1199	0.1496	1
N(5)	−0.2558	0.1241	0.1777	1
N(6)	0.6479	−0.0797	0.3459	1
C(1)	0.2393	0.1315	0.0684	1
C(2)	0.1370	−0.1450	0.4087	1
C(3)	0.2567	0.1651	0.3834	1
C(4)	0.1284	−0.1766	0.0920	1
C(5)	−0.1237	0.1064	0.2229	1
C(6)	−0.3221	0.0645	0.1043	1
C(7)	−0.3506	0.2042	0.2045	1
C(8)	0.5134	−0.0630	0.3271	1
C(9)	0.6986	−0.1570	0.2902	1
C(10)	0.7489	−0.0485	0.4228	1

Table S2. Fractional atomic coordinates for $Co[Au(CN)_2]_2(pyridine)_2$.

Atom	z	y	z		Occupancy
Au(1)	0.2471	0.2188	−0.0358		1
Au(2)	0.1489	−0.2322	0.5018		1
Co(1)	0.1777	0.0012	0.2447		1
N(10)	−0.0735	0.0292	0.2144		1
N(11)	0.3744	−0.0197	0.2715		1
N(1)	0.2384	0.0807	0.1293		1
N(2)	0.1418	−0.0901	0.3506		1
N(3)	0.2324	0.1077	0.3269		1
N(4)	0.1418	−0.1199	0.1496		1
C(1)	0.2393	0.1315	0.0684		1
C(2)	0.1370	−0.1450	0.4087		1
C(3)	0.2567	0.1651	0.3834		1
C(4)	0.1284	−0.1766	0.0920		1
C(10)	0.4348	−0.1028	0.2514		1
C(11)	0.5877	−0.1213	0.2740		1
C(12)	0.6829	−0.0552	0.3174		1
C(13)	0.6202	0.0287	0.3398		1
C(14)	0.4657	0.0458	0.3141		1
C(20)	−0.1702	−0.4627	−0.3245		1
C(21)	−0.3247	−0.4777	−0.3411		1
C(22)	−0.3841	−0.5613	−0.3187		1
C(23)	−0.2852	−0.6283	−0.2770		1
C(24)	−0.1295	−0.6114	−0.2632		1

Table S3. Unit cell parameters for $Co[Au(CN)_2]_2$.

	$Co[Au(CN)_2]_2$
Sample	powder
Crystal system	tetragonal
Space group	$P\bar{4}b2$
a, Å	6.7968(17)
c, Å	8.4651(3)
V, Å^3	391.47

Table S4. Fractional atomic coordinates for $Co[Au(CN)_2]_2$.

Atom	*x*	*y*	*z*
Au(1)	0.6733	0.1733	0.5000
Co(1)	0.0000	0.0000	0.0000
C(1)	0.1034	0.2625	0.2771
N(1)	0.0582	0.2164	0.1499

Figure S1. Comparison between the powder X-ray diffractograms for γ-$Zn[Au(CN)_2]_2$ (*Zn*, red), $Co[Au(CN)_2]_2$ (*Co*, blue) and the simulated diffractogram for $Co[Au(CN)_2]_2$ predicted by its structural model (*Co**, black). The * on the blue *Co* spectrum corresponds to a small amount of $Co(\mu\text{-}OH_2)_2[Au(CN)_2]_2$ impurity.

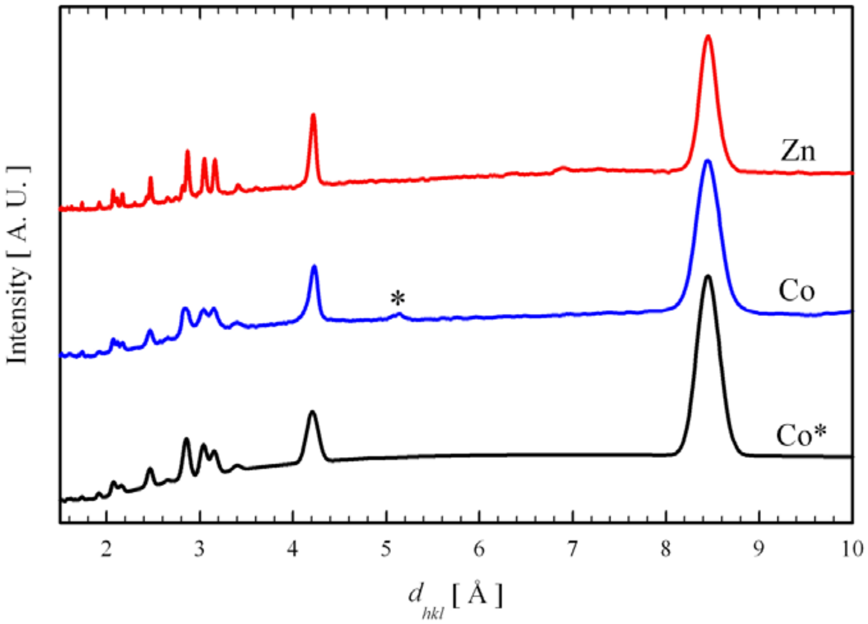

© 2012 by the authors; licensee MDPI, Basel, Switzerland. This article is an open access article distributed under the terms and conditions of the Creative Commons Attribution license (http://creativecommons.org/licenses/by/3.0/).
